# Phenylindanes in Brewed Coffee Inhibit Amyloid-Beta and Tau Aggregation

**DOI:** 10.3389/fnins.2018.00735

**Published:** 2018-10-12

**Authors:** Ross S. Mancini, Yanfei Wang, Donald F. Weaver

**Affiliations:** ^1^Department of Fundamental Neurobiology, Krembil Research Institute, University Health Network, Toronto, ON, Canada; ^2^Department of Chemistry, University of Toronto, Toronto, ON, Canada; ^3^Department of Medicine, University of Toronto, Toronto, ON, Canada

**Keywords:** Alzheimer’s, Parkinson’s, protein misfolding, aggregation inhibition, coffee, caffeine, phenylindane, neuroprotective

## Abstract

Coffee consumption has been correlated with a decreased risk of developing Alzheimer’s disease (AD) and Parkinson’s disease (PD), but the mechanism by which coffee may provide neuroprotection in humans is not fully understood. We hypothesized that compounds found in brewed coffee may elicit neuroprotective effects by inhibiting the aggregation of amyloid-beta (Aβ) and tau (AD) or α-synuclein (PD). Three instant coffee extracts (light roast, dark roast, decaffeinated dark roast) and six coffee components [caffeine (**1**), chlorogenic acid (**2**), quinic acid (**3**), caffeic acid (**4**), quercetin (**5**), and phenylindane (**6**)] were investigated for their ability to inhibit the fibrillization of Aβ and tau proteins using thioflavin T (ThT) and thioflavin S (ThS) fluorescence assays, respectively. Inhibition of Aβ and α-synuclein oligomerization was assessed using ELISA assays. All instant coffee extracts inhibit fibrillization of Aβ and tau, and promote α-synuclein oligomerization at concentrations above 100 μg/mL. Dark roast coffee extracts are more potent inhibitors of Aβ oligomerization (IC_50_ ca. 10 μg/mL) than light roast coffee extract (IC_50_ = 40.3 μg/mL), and pure caffeine (**1**) has no effect on Aβ, tau or α-synuclein aggregation. Coffee components **2**, **4,** and **5** inhibit the fibrillization of Aβ at 100 μM concentration, yet only **5** inhibits Aβ oligomerization (IC_50_ = 10.3 μM). **1**–**5** have no effect on tau fibrillization. Coffee component **6**, however, is a potent inhibitor of both Aβ and tau fibrillization, and also inhibits Aβ oligomerization (IC_50_ = 42.1 μM). Coffee components **4** and **5** promote the aggregation of α-synuclein at concentrations above 100 μM; no other coffee components affect α-synuclein oligomerization. While the neuroprotective effect of coffee consumption is likely due to a combination of factors, our data suggest that inhibition Aβ and tau aggregation by phenylindane **6** (formed during the roasting of coffee beans, higher quantities found in dark roast coffees) is a plausible mechanism by which coffee may provide neuroprotection. The identification of **6** as a dual-inhibitor of both Aβ and tau aggregation is noteworthy, and to our knowledge this is the first report of the aggregation inhibition activity of **6**.

## Introduction

Coffee is one of the most widely consumed beverages worldwide, with approximately 500 billion cups consumed annually (Butt and Sultan, 2011). Coffee drinks are available in a variety of different flavors, roasting levels, and also with or without caffeine. More recently, coffee has attracted interest in the medical community due to a growing number of epidemiological studies and meta-analyses which have correlated coffee consumption with a reduced risk of developing diabetes mellitus, various cancers and neurodegenerative disorders such as Alzheimer’s disease (AD) and Parkinson’s disease (PD) (Butt and Sultan, 2011). It is generally accepted that caffeine and caffeinated coffee elicit short-term improvements in alertness, attention and memory (Butt and Sultan, 2011), but the ability to protect against age-related cognitive decline is less clear. A 2010 systematic review and meta-analysis by Santos et al. (2010) suggests that a positive correlation between coffee consumption and dementia prevention is apparent, but inconsistencies in study design and methodology make interpretation of experimental results complicated (Costa et al., 2010). A more recent study by Mirza et al. (2014) claim that there is no correlation between coffee consumption and incident dementia, attributing the perceived neuroprotective benefits to a reverse causality effect.

The major psychoactive component of coffee is caffeine (**1**), a xanthine alkaloid that readily crosses the blood-brain barrier (BBB). It is known that caffeine content in coffee is highly variable, and depends on the type of coffee bean (Arabica *versus* Robusta), roasting method (light roast *versus* dark roast) and extraction method (drip *versus* boiled) employed. For example, a standard cup of drip coffee contains on average 70—80 mg caffeine per 150 mL (Barone and Roberts, 1996), whereas espresso coffees contain approximately 950 mg caffeine per 150 mL (Ludwig et al., 2014). However, given the differences in serving size between a typical 8 oz cup of drip coffee and 1 oz espresso shot, the overall caffeine intake is similar. Caffeine is also highly bioavailable, with nearly 100% absorption of caffeine occurring 45 min post-ingestion (Blanchard and Sawers, 1983). These favorable drug-like properties have prompted research efforts into the use of caffeine to treat cognitive decline associated with AD (Arendash et al., 2006, 2009) and PD (Chen et al., 2001; Xu et al., 2010). However, it has recently been suggested that chronic caffeine administration may exacerbate behavioral and psychological symptoms of patients with dementia (anxiety-related behaviors), and could interfere with any potential cognitive benefits of caffeine consumption (Baeta-Corral et al., 2018).

In addition to caffeine, coffee also contains a variety of polyphenolic acids known collectively as chlorogenic acids, as well as various flavinoids, tannins and melanoidans. In fact, transgenic mouse models of AD that were administered crude caffeine – the byproduct of coffee’s decaffeination process, containing a variety of other phytochemicals in addition to caffeine – exhibited less memory impairment and lower hippocampal levels of Aβ peptide and Aβ plaques versus mice treated with pure caffeine (Chu et al., 2012). It has even been suggested that caffeine may have synergistic effects with other components in coffee to produce the overall neuroprotective effect (Cao et al., 2011). Our lab has an interest in identifying naturally occurring small molecules which can inhibit the aggregation of amyloidogenic proteins; the formation of protein aggregates in the brain (in particular pre-fibrillar oligomers) is known to be neurotoxic (Ono, 2017, 2018), and inhibition of the aggregation process is an attractive strategy for the treatment of neurodegenerative disorders. As such, there is a need for the identification of drug-like molecules capable of preventing the aggregation of amyloidogenic proteins associated with AD (Aβ/tau) and PD (α-synuclein) to spur drug discovery projects. It is worth noting, however, that some small molecule inhibitors of protein aggregation exhibit their effects by stabilizing smaller oligomeric species (Dedmon et al., 2005; Taniguchi et al., 2005; Masuda et al., 2006; Zhu et al., 2013; Matos et al., 2017), which have the potential to be neurotoxic (Caruso et al., 2017).

**FIGURE 1:**
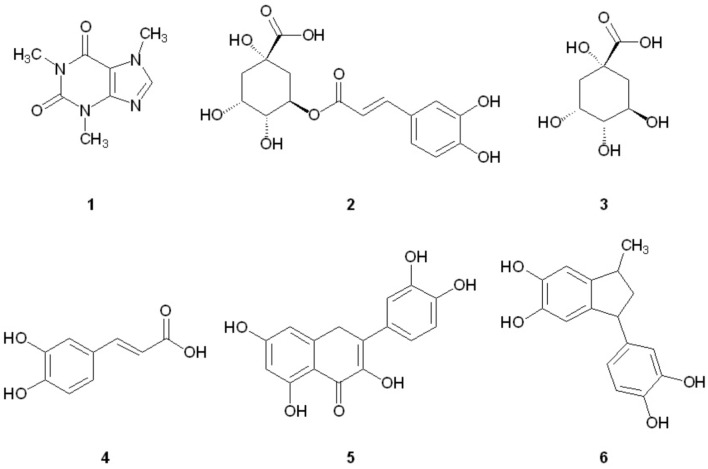
Structures of coffee components investigated for aggregation inhibition activity.

We have recently reported that components of Canadian maple syrup can reduce the aggregation of amyloidogenic Aβ and tau (Hawco et al., 2016), and we were curious if caffeine or other components found in brewed coffee elicit neuroprotective effects through a similar mechanism. Herein we report our investigation into the effects of coffee on the aggregation of misfolded proteins associated with dementia to determine if inhibition of protein aggregation is a viable mechanism of neuroprotection associated with coffee consumption. We show that a number of polyphenolic compounds found in brewed coffee (**Figure 1**) can alter the aggregation profile of Aβ, tau and α-synuclein. Of the coffee components investigated, pyrolysis product **6** is found to be a potent inhibitor of both Aβ and tau aggregation, and is the only component tested capable of inhibiting tau aggregation. A related series of indanedione analogs are also known to inhibit Aβ fibrillization (Catto et al., 2010), but tau aggregation was not investigated in this report. To our knowledge, this is the first report of the aggregation inhibition activity of **6** for Aβ, tau and α-synuclein.

## Materials and Methods

### Coffee Extracts and Coffee Components

Starbucks brand 100% Arabica instant coffee (Starbucks Via Instant^TM^) was obtained in three different varieties: light roast, dark roast and decaffeinated dark roast. Stock solutions of coffee extracts were prepared in water that was micropore filtered and deionized (MilliQ^®^ Advantage A10, Millipore Sigma) at a concentration of 10.0 mg/mL for *in vitro* screening. Caffeine (**1**) was purchased from BDH biochemical (Mississauga, ON, Canada). Chlorogenic acid (**2**), quinic acid (**3**), caffeic acid (**4**) and quercetin (**5**) were purchased from Combi-Blocks (San Diego, CA, United States). Phenylindane (**6**) was prepared as outlined below, according to modified literature procedure (Stadler et al., 1996). Stock solutions of compounds **1**–**4** and **6** were prepared in MilliQ^®^ water at a concentration of 25.0 mM for *in vitro* screening. Stock solution of compound **5** was prepared in DMSO (due to low solubility in water) at 25.0 mM for *in vitro* screening.

### Chemical Synthesis of Phenylindane (6)

Flash chromatography was performed on RediSep columns using an Isco CombiFlash system from Teledyne Isco (Lincoln, NE, United States). Thin-layer chromatography (TLC) was performed using Merck silica gel 60 F_254_ aluminum-backed plates, and products were visualized by UV irradiation at 254 nm. Solvents were purchased from Sigma-Aldrich (Oakville, ON, Canada) or Fisher Scientific (Markham, ON, Canada). Deionized water was acquired from an in-house supply. Proton nuclear magnetic resonance (^1^H NMR) spectra and carbon nuclear magnetic resonance (^13^C NMR) spectra were recorded on a 500 MHz Agilent DD2 spectrometer (Santa Clara, CA, United States). Chemical shifts for protons are reported in parts per million (ppm) downfield from tetramethylsilane and are referenced to residual protium in the NMR solvent (CD_3_OD: δ 3.31). Chemical shifts for carbon are reported in ppm downfield from tetramethylsilane and are referenced to the carbon resonances of the solvent (CD_3_OD: δ 49.0). Data are represented as follows: chemical shift (δ, ppm); multiplicity (s, singlet; d, doublet; dd, doublet of doublets; ddd, doublet of doublet of doublets; q, quartet; m, multiplet); integration; coupling constant (*J*, Hz), proton assignment. Proton resonances were assigned based on 2-D COSY, HSQC, and HMBC experiments. Mass spectra were recorded using electron spray ionization (ESI^+^) with a Xevo TQ-XS Tandem Triple Quadrupole Mass Spectrometer (Waters brand, Taunton, MA, United States). High-performance liquid chromatography (HPLC) was performed using a Waters Acquity UPLC (2.1 × 50 mm, 1.7 μm particle size) using gradient elution [acetonitrile/0.100% (*v*/*v*) formic acid in water/0.100% (*v*/*v*) formic acid] at a flow rate of 0.600 mL/min. UV detection was carried out at a wavelength of 254 nm.

Phenylindane (**6)** was prepared according to modified literature procedure (Stadler et al., 1996): caffeic acid (**4**) (5.00 g, 27.8 mmol) was suspended in a solution of 2.00 N sulfuric acid (100 mL) and heated to reflux. The solution was stirred at reflux for 4 h and then allowed to cool to room temperature. The organic compounds were extracted by washing of the aqueous phase three times with diethyl ether. The organic extracts were combined and dried over anhydrous magnesium sulfate, filtered and concentrated under reduced pressure to give a purple solid. The crude material was purified by flash column chromatography on silica gel (gradient elution, 10–50% diethyl ether in hexanes). Product fractions were identified by treating aliquots of the collected fractions with a small amount of 1.00 M sodium hydroxide aqueous solution; a deep purple color is observed for phenylindane-rich fractions. Product fractions were collected and the solvent was removed under reduced pressure to yield 665 mg of a brown/orange amorphous solid which contained ca. 25% of an unidentified byproduct. This material was further purified using reverse-phase flash column chromatography on C18 silica gel (1:9 acetonitrile:water). Product fractions were combined and washed three times with methyl *tert*-butyl ether (MTBE). The organic extracts were combined and dried over anhydrous magnesium sulfate, filtered and concentrated under reduced pressure to give an orange amorphous solid. This material was once again purified by flash column chromatography on silica gel (6:4 MTBE:hexanes) to yield pure **6** as an orange amorphous solid (204 mg, 2.69%), isolated as a mixture of stereoisomers (1.00:1.87 *cis*:*trans*). NMR data are consistent with previous literature reports (Stadler et al., 1996). TLC *R*_F_ = 0.65 (diethyl ether). *cis*-Isomer: ^1^H NMR (500 MHz, CD_3_OD): δ (ppm) 6.69 (d, 1H, *J* = 8.0 Hz, H-5′), 6.63 (d, 1H, *J* = 0.9 Hz, H-7), 6.60 (d, 1H, *J* = 2.1 Hz, H-2′), 6.53 (ddd, 1H, *J* = 8.1, 2.1, 0.4 Hz, H-6′), 6.27 (d, 1H, *J* = 1.0 Hz, H-4), 3.94 (dd, 1H, *J* = 10.6, 7.1 Hz, H-1), 3.01 (m, 1H, H-3), 2.58 (dd, 1H, *J* = 12.0, 6.9 Hz, H-2), 1.43 (dd, 1H, *J* = 12.0, 10.4 Hz, H-2), 1.28 (d, 1H, *J* = 6.7 Hz, CH_3_). ^13^C NMR (125 MHz, CD_3_OD): δ (ppm) 146.2, 145.2, 144.9, 144.6, 141.1, 139.7, 138.7, 120.7, 116.1, 116.1, 112.4, 110.5, 51.0, 48.4, 39.0, 20.1. *trans*-Isomer: ^1^H NMR (500 MHz, CD_3_OD): δ (ppm) 6.65 (d, 1H, *J* = 8.0 Hz, H-5′), 6.64 (s, 1H, H-7), 6.49 (d, 1H, *J* = 2.1 Hz, H-2′), 6.44 (dd, 1H, *J* = 8.1, 2.2 Hz, H-6′), 6.38 (d, 1H, *J* = 0.8 Hz, H-4), 4.13 (dd, 1H, *J* = 8.0, 5.7 Hz, H-1), 3.19 (m, 1H, H-3), 2.14 (ddd, 1H, *J* = 12.5, 7.6, 5.9 Hz, H-2), 2.05 (ddd, 1H, *J* = 12.5, 8.1, 5.5 Hz, H-2), 1.17 (d, 1H, *J* = 6.7 Hz, CH_3_). ^13^C NMR (125 MHz, CD_3_OD): δ (ppm) 146.1, 145.4, 145.1, 144.3, 141.5, 139.6, 138.7, 120.1, 116.1, 115.7, 112.7, 111.0, 50.0, 46.7, 38.9, 21.3. HRMS (ESI, *m*/*z*) Calculated for [C_16_H_16_O_4_] (M)^+^ 272.1043; Found 272.1047. Calculated for [C_16_H_15_O_4_] (M-H)^+^ 271.0965; Found: 271. 0970.

### β-Amyloid ThT Aggregation Assay

This assay was adapted from LeVine (1993). Aβ_1-40_ (>95%) was purchased from AnaSpec (Freemont, CA, United States) and stored at -80°C. All other reagents were of the highest available purity, purchased from Sigma-Aldrich (Oakville, ON, Canada), and used without further purification. All water used in the assays was micropore filtered and deionized (MilliQ^®^). Aβ_1-40_ (1.00 mg) was dissolved in hexafluoro-2-propanol (HFIP) and sonicated for 30 min to disassemble any pre-formed aggregates. HFIP was removed using a stream of argon gas prior to dissolution of Aβ_1-40_ in 1.00 mL Tris base (20.0 mM, pH 10.0) using vortex and 10 min sonication. The solution was then further diluted with 4.70 mL of Tris base followed by adjusting to pH 7.40 using concentrated hydrochloric acid and then filtered using a 0.200 μm syringe filter. The pretreated Aβ_1-40_ was diluted with an equal volume (5.70 mL) of 8.00 μM ThT in Tris (20.0 mM, pH 7.40, 300 mM NaCl) and 200 μL aliquots of this solution [20.0 μM Aβ_1-40_ and 4.00 μM ThT in Tris (20.0 mM, pH 7.40, 150 mM NaCl)] were added to wells of a black polystyrene 96-well plate. 4.00 μL of test compound solutions at various concentrations were added to each well. Each sample was performed in triplicate and MilliQ^®^ water alone served as a vehicle control. Plates were sealed and incubated in a microplate reader (Tecan Genios) at 37.0°C with fluorescence measurements recorded (λ_ex_ = 450 nm, λ_em_ = 480 nm) every 15 min after first being shaken at high intensity for 15 s and then allowed to settle for 10 s before each reading.

### β-Amyloid Oligomerization Assay

This assay was adapted from LeVine (2006). Biotinylated Aβ_1-42_ was purchased from AnaSpec (Freemont, CA, United States). An ELISA plate (Costar 9018) was coated with 50.0 μL of a stock solution containing 1.00 μg/mL NeutrAvidin in sodium phosphate buffer (10.0 mM, pH 7.50). The plate was sealed and stored at 4°C overnight prior to blocking for 2 h at room temperature with 200 μL/well of OFB-T buffer [20.0 mM sodium phosphate, 150 mM NaCl, pH 7.50, 0.100% (*v*/*v*) Tween 20]. Then, 20.0 μL of Aβ_1-42_ stock solution (0.100 mg/mL) was treated with HFIP and dried under a stream of argon. One hundred microliter of trifluoroacetic acid (TFA) was added to the tube and the sample was dissolved using a vortex mixer prior to drying under a stream of argon. HFIP was added and dried under a stream or argon to remove residual TFA. The biotinylated Aβ_1-42_ was then dissolved in 870 μL of DMSO, and 2.00 μL of the solution was added to each well of a 96-well polypropylene plate (Costar 3365) followed by 100 μL of test compound diluted in OFB-T buffer (various concentrations). The plate was incubated for 1 h at room temperature without shaking, and then stopped by the addition of 50.0 μL of 0.300% (*v*/*v*) Tween 20 in MilliQ^®^ water. 50.0 μL of the biotinylated Aβ_1-42_/compound solutions was added to each well of the NeutrAvidin^TM^-coated plate (after removing blocking solution) and the plate was sealed and incubated for 2 h with shaking at 150 rpm. The plate was washed three times with TBST solution [20.0 mM Tris-HCl, 34.0 mM sodium chloride, pH 7.50, 0.100% (*v*/*v*) Tween 20], then 50.0 μL of Streptavidin-HRP (1:20,000) in OFB-T buffer was added and the plate was sealed and incubated for 1 h with shaking at 150 rpm. The plate was again washed three times with TBST, followed by addition of 100 μL of tetramethylbenzidine/H_2_O_2_ substrate solution to each well. The reaction was stopped after 10–30 min by the addition of 100 μL of 2.00% (*v*/*v*) aqueous sulfuric acid prior to reading absorbance at 450 nm in a plate reader.

### Preparation of Tau Protein

Human tau_40_ (tau) cDNA was amplified by polymerase chain reaction (PCR) with tau forward primer (5′-GCTGAGCCCCGCCAGGAGTTCG-3′) and tau reverse primer (5′-TCACAAACCCTGCTTGGCCAGG-3′) using Taq DNA polymerase (ThermoFisher Scientific, Waltham, MA, United States) and was cloned into Champion pET SUMO vector (ThermoFisher Scientific, Waltham, MA, United States) by using TA cloning. After transforming into TOP10 *E. coli* strain, colonies with correct orientation were selected by colony PCR with T7 reverse primer (5′-TAGTTATTGCTCAGCGGTGG-3′) and tau internal forward primer (5′-CGCATGGTCAGTAAAAGCAAA-3′). Plasmid DNAs were prepared, sequenced and the one with correct sequence was selected. pET SUMO tau vector was then transformed into BL21(DE3) *E. coli* strain for the expression of the gene. BL21(DE3) *E. coli* with pET SUMO tau vector were grown in media71757 (Overnight Express^TM^ Instant LB Medium – Novagen with kanamycin). Expression was maintained for 7 h before harvesting the cells by centrifugation. Harvested *E. coli* were lysed by sonication in the presence of 1.00% (*v*/*v*) Triton X-100/50.0 mM Tris-HCl/pH 7.40/500 mM NaCl/EDTA-free protease inhibitors cocktail (Roche, Germany) in ice. Soluble lysate was recovered after removing insoluble debris by centrifugation at 20,000 × *g*. SUMO-tau with 6 × His tag was purified by elution through Talon IMAC resin (Clonetech, Mountain View, CA, United States) and the eluent was dialyzed against 50.0 mM Tris-HCl/pH 7.40/150 mM NaCl/1.00 mM dithiothreitol/0.200% (*v*/*v*) NP40. 6 × His-SUMO tag was cleaved using SUMO protease and tag-free tau protein was recovered by elution through Talon IMAC column. The tag-free tau protein was further purified by preparative reversed-phase HPLC using a C18 column (150 × 21.2 mm, 5.00 μm particle size) using gradient elution [1–30% acetonitrile/0.100% (*v*/*v*) formic acid in MilliQ^®^ water/0.100% (*v*/*v*) formic acid] at a flow rate of 4.00 mL/min. UV detection was carried out at a wavelength of 220 nM.

### Tau ThS Aggregation Assay

This assay was adapted from Friedhoff et al. (1998). A 4.00 μM solution of tau in 5.00 μM ThS in Tris (50.0 mM, pH 7.40, 10.0 μg/mL heparin, 1.00 mM dithiothreitol, 50.0 μM sodium azide) was prepared fresh for each experiment and 200 μL aliquots were added to wells of a black polystyrene 96-well plate. 4.00 μL of compound solutions at various concentrations were added to each well. Each sample was performed in triplicate and MilliQ^®^ water alone served as a vehicle control. Plates were sealed and incubated in a microplate reader (Tecan Genios) at 37°C with fluorescence measurements recorded (λ_ex_ = 450 nm, λ_em_ = 480 nm) every 15 min.

### α-Synuclein Oligomerization Assay

This assay was adapted from El-Agnaf et al. (2004). Capture antibody (α-synuclein 211) and detection antibody (α-synuclein 211, HRP) were purchased from Santa Cruz (Freemont, CA, United States) and stored at 4°C. α-Synuclein was purchased from rPeptide (Watkinsville, GA, United States) and stored at -80°C. A 0.100 mg/mL stock solution of α-synuclein in DMSO was prepared, and 1.80 μg of α-synuclein was diluted with 5.00 mL of phosphate buffered saline (PBS), mixed well using vortex and 50.0 μL aliquots were added to each well of a low-binding plate (Costar). Compound solutions of various concentrations were prepared in PBS, and 50.0 μL aliquots were added to each well of the plate containing α-synuclein. The plate was shaken at 450 rpm for 45 s at 20°C using Eppendorf plate shaker, and then incubated for 16 h at room temperature without shaking. The reaction was stopped by the addition of 50.0 μL of 0.300% (*v*/*v*) Tween 20 in MilliQ^®^ water and gently mixed. 50.0 μL of the capture antibody in 10.0 mM sodium phosphate/pH 7.50 was added to each well of a high-binding ELISA plate (Costar) and allowed to sit overnight at 4°C. The plate was blocked by addition of 250 μL of TBST with 2.00% fatty acid-free bovine serum albumin at room temperature for 1–2 h. The plate was washed three times with TBST after blocking, then 50–100 μL of the test compound/α-synuclein solution was added to each well and the plate was sealed and incubated at room temperature for 2 h. The plate was then washed, and 50.0 μL of the detection antibody (1:1000 in TBST) was added. The plate was sealed and incubated at room temperature for 1 h followed by washing three times with TBST. 100 μL of tetramethylbenzidine/H_2_O_2_ substrate solution was added to each well. Reaction was stopped after 25–30 min with the addition of 100 μL of 0.118 M aqueous sulfuric acid prior to reading absorbance at 450 nm in a plate reader.

### Statistical Analysis

Data analysis was performed using GraphPad Prism software (version 7.04; GraphPad Software, San Diego, CA, United States). Differences were considered statistically significant at *P* < 0.05. Fibrillization data were expressed as mean ± standard deviation; % inhibition was calculated relative to vehicle control sample containing no analyte (taken as 0% inhibition). Group differences in % inhibition were assessed using two-way ANOVAs to explore factors of roasting level (light roast *versus* dark roast) and caffeine content (caffeinated dark *versus* decaffeinated dark) across each concentration level; *post-hoc* pairwise comparisons were performed using Sidak’s tests. Inhibitory effects of coffee components **2**–**6** were analyzed using one-way ANOVA with *post-hoc* Dunnett’s test.

Oligomerization data were expressed as mean IC_50_ ± standard deviation; IC_50_ values were extrapolated by fitting to a dose response curve using least squares regression (*R*^2^ > 0.9). Group differences in IC_50_ values were analyzed using unpaired two-tailed *t*-tests for each factor (roasting level or caffeine content). Bonferroni correction was applied for running multiple comparisons (*P* = 0.05/2 tests).

## Results

### Coffee Extracts and Caffeine

**FIGURE 2:**
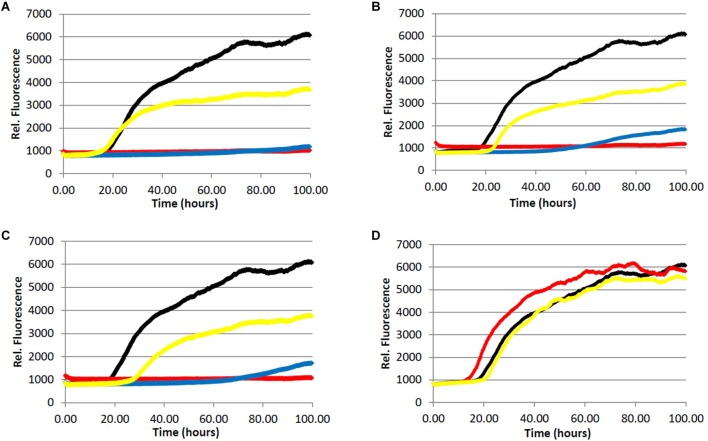
Fibrillization of Aβ peptide. 20.0 μM Aβ_1-40_ in Tris buffer (20.0 mM, pH 7.4, 150 mM NaCl) was incubated at 37.0°C in the presence of 4.0 μM ThT and variable concentration of analyte. (red) = 200 μg/mL, (blue) = 40.0 μg/mL, (yellow) = 5.0 μg/mL, (black) = 0.0 μg/mL (vehicle control). **(A)** Light roast instant coffee extract. **(B)** Dark roast instant coffee extract. **(C)** Decaffeinated dark roast coffee extract. **(D)** Caffeine **1**.

**FIGURE 3:**
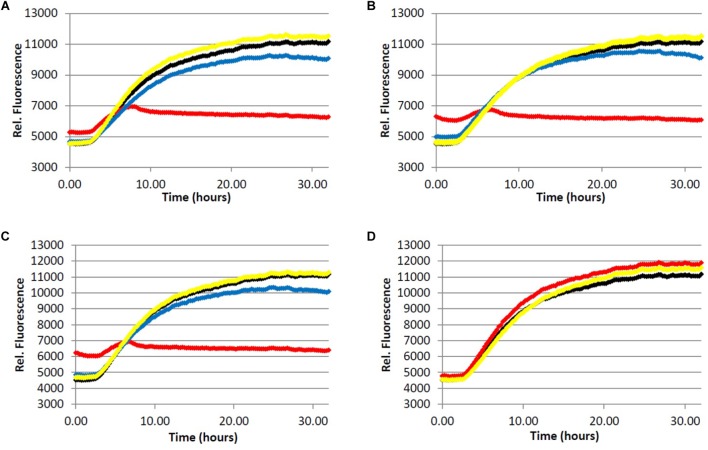
Fibrillization of tau protein. 4.0 μM tau in Tris buffer (50.0 mM, pH 7.4, 10.0 μg/mL heparin, 1.0 mM dithiothreitol, 50.0 mM sodium azide) was incubated at 37.0°C in the presence of 5.0 μM ThS and variable concentration of analyte. (red) = 200 μg/mL, (blue) = 40.0 μg/mL, (yellow) = 5.0 μg/mL, (black) = 0.0 μg/mL (vehicle control). **(A)** Light roast instant coffee extract. **(B)** Dark roast instant coffee extract. **(C)** Decaffeinated dark roast coffee extract. **(D)** Caffeine **1**.

We began by assessing the aggregation inhibition activity of instant coffee extracts. We chose to investigate three different types of coffee – light roast, dark roast, and decaffeinated dark roast. The effect of caffeine content would be assessed by comparing the activity of caffeinated and decaffeinated dark roast coffee extracts. Further, since it is known that different levels of roasting affect the composition of the coffee brew (Farah et al., 2005; Jaiswal et al., 2012), comparison of light *versus* dark roast coffee extracts was also performed. The ability of the coffee extracts to inhibit the formation of fibrillar aggregates was investigated using a ThT fluorescence assay for Aβ (**Figure 2**) and ThS fluorescence assay for tau (**Figure 3**).

All three coffee extracts inhibited the fibrillization of both Aβ and tau proteins at 200 μg/mL concentration, with moderate levels of inhibition maintained for Aβ fibrils at 5 μg/mL (**Table 1**). A small but measurable level of inhibition was observed for tau fibrillization at 40 μg/mL coffee extract (**Table 2**). For Aβ fibrillization, no main effects of roast level (light *versus* dark) or caffeine content (dark roast *versus* decaffeinated dark roast) were observed. For tau fibrillization, an effect of roast level was observed (*P* = 0.0059), but only at concentrations of 200 μg/mL (*P* < 0.0001); caffeine content had no effect. We were surprised to find that caffeine content did not influence aggregation inhibition, and thus performed a *post-hoc* analysis of pure caffeine (**1**) (one-way ANOVA). No effect on fibril growth was observed relative to the vehicle control, consistent with the results for caffeinated *versus* decaffeinated coffee extracts.

**Table 1:** Inhibition of Aβ fibril formation by coffee extracts in ThT fluorescence assay.^a^

Concentration (μg/mL)	Light roast coffee extract (% inhibition)	Dark roast coffee extract (% inhibition)	Decaffeinated dark roast coffee extract (% inhibition)	Caffeine (1)^b^ (% inhibition)
200	98.5 ± 1.0	100.1 ± 1.8	100.5 ± 1.5	-5.4 ± 3.5
40.0	96.6 ± 1.0	88.4 ± 1.6	94.9 ± 1.4	–
5.0	46.6 ± 2.2	46.5 ± 2.2	47.4 ± 2.5	5.7 ± 3.0


*^*a*^% inhibition calculated relative to vehicle control sample (taken as 0% inhibition), and is reported as the mean ± standard deviation of three separate experiments. ^*b*^*Post-hoc* investigation of pure caffeine (**1**): no significant effect relative to vehicle control (one-way ANOVA with *post-hoc* Dunnett’s test).*

**Table 2:** Inhibition of tau fibril formation by coffee extracts in ThS fluorescence assay.^a^

Concentration (μg/mL)	Light roast coffee extract (% inhibition)	Dark roast coffee extract (% inhibition)	Decaffeinated dark roast coffee extract (% inhibition)	Caffeine (1)^c^ (% inhibition)
200	86.2 ± 1.0	102.6 ± 2.4	95.8 ± 2.1	-4.2 ± 3.4
40.0	17.0 ± 2.4	17.7 ± 2.4	17.6 ± 2.3	–
5.0^b^	-4.5 ± 3.2	-5.5 ± 3.3	-0.9 ± 2.9	-4.0 ± 3.2


*^*a*^% inhibition calculated relative to vehicle control sample (taken as 0% inhibition), and is reported as the mean ± standard deviation of three separate experiments. ^*b*^No significant effect relative to vehicle control. ^*c*^*Post-hoc* investigation of pure caffeine (**1**): no significant effect relative to vehicle control (one-way ANOVA with *post-hoc* Dunnett’s test).*

The coffee extracts were also investigated for their ability to inhibit oligomerization of Aβ and α-synuclein: IC_50_ values for coffee extracts and caffeine are shown in **Table 3**. All coffee extracts showed potent aggregation inhibition activity for Aβ and displayed pro-aggregation effects for α-synuclein at concentrations above 100 μg/mL. Furthermore, the dark roast coffee extract showed more potent aggregation inhibition activity for Aβ oligomerization than the light roast coffee extract (*P* < 0.001), and decaffeinated dark roast coffee extract displayed almost identical potency to the caffeinated dark roast coffee extract (*P* > 0.9). Pure caffeine (**1**) failed to inhibit the oligomerization of Aβ or α-synuclein, and no measurable IC_50_ values could be obtained.

**Table 3:** IC_50_ values for coffee extracts and caffeine in oligomer assays.^a^

Extract	Aβ IC_50_ (μg/mL)	α-synuclein IC_50_ (μg/mL)
Light roast coffee extract	40.3 ± 4.6	pro-aggregate
Dark roast coffee extract	9.5 ± 1.7	pro-aggregate
Decaffeinated dark roast coffee extract	9.4 ± 1.5	pro-aggregate
Caffeine (**1**)	N.D.	N.D.


*^*a*^Data are reported as the mean ± standard deviation of three separate experiments. Pro-aggregate effect observed at concentrations >100 μg/mL. N.D. = not determined (>200 μM).*

### Coffee Components

**FIGURE 4:**
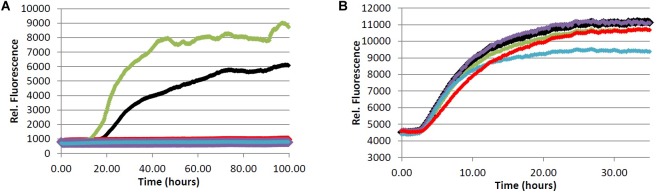
**(A)** Fibrillization of Aβ peptide. 20.0 μM Aβ_1-40_ in Tris buffer (20.0 mM, pH 7.4, 150 mM NaCl) was incubated at 37.0°C in the presence of 4.0 μM ThT and 100 μM of analyte. **(B)** Fibrillization of tau protein. 4.0 μM tau in Tris buffer (50.0 mM, pH 7.4, 10.0 μg/mL heparin, 1.0 mM dithiothreitol, 50.0 mM sodium azide) was incubated at 37.0°C in the presence of 5.0 μM ThS and 100 μM of analyte. (red) = chlorogenic acid (**2**), (green) = quinic acid (**3**), (purple) = caffeic acid (**4**), (blue) = quercetin (**5**), (black) = no compound (vehicle control).

**FIGURE 5:**
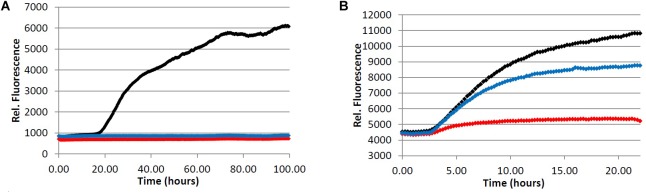
Inhibition of fibril formation by phenylindane **6**. **(A)** Fibrillization of Aβ peptide. 20.0 μM Aβ_1-40_ in Tris buffer (20.0 mM, pH 7.4, 150 mM NaCl) was incubated at 37.0°C in the presence of 4.0 μM ThT and variable concentration of analyte. **(B)** Fibrillization of tau protein. 4.0 μM tau in Tris buffer (50.0 mM, pH 7.4, 10.0 μg/mL heparin, 1.0 mM dithiothreitol, 50.0 mM sodium azide) was incubated at 37.0°C in the presence of 5.0 μM ThS and variable concentration of analyte. (red) = 100 μM, (blue) = 20.0 μM, (black) = 0.0 μM (vehicle control).

In an effort to determine the active component(s) in the coffee extracts responsible for the observed aggregation inhibition activity, we assayed the compounds shown in **Figure 1** as representative examples of coffee’s individual components (for a rationale of how the components were chosen, see Discussion section below). All compounds were able to inhibit the formation of Aβ fibrils at 100 μM, with the exception of quinic acid (**3**) which was found to promote the growth of Aβ fibrils relative to the control sample (**Figures 4A** and **5A**). Tau fibrillization (**Figures 4B** and **5B**) was unaffected by compounds **2**–**4** at 100 μM, but a measurable inhibitory effect was observed for **5** and **6** (23.7 and 95.2% inhibition, respectively). Given the potent activity of **6** toward both Aβ and tau, phenylindane (**6**) was rescreened in the fibrillization assays at a reduced concentration of 20 μM; Aβ fibrillization remained completely inhibited (**Figure 5A**), and modest levels of tau inhibition were maintained (**Figure 5B**). Calculated values for % inhibition of Aβ and tau fibrillization by **2**–**6** are shown in **Table 4**.

**Table 4:** Inhibition of Aβ and tau fibril formation by **2**–**6** in ThT and ThS fluorescence assays.^a^

Coffee component	Aβ fibril inhibition (%)	Tau fibril inhibition (%)
Chlorogenic acid (**2**)	97.7 ± 0.7	3.4 ± 2.9^b^
Quinic acid (**3**)	-48.8 ± 2.8	6.7 ± 3.0^b^
Caffeic acid (**4**)	99.4 ± 0.9	-2.6 ± 3.4^b^
Quercetin (**5**)	97.9 ± 0.4	23.7 ± 2.4
Phenylindane (**6**)	99.0 ± 0.4	95.2 ± 1.7
Phenylindane (**6**)**^c^**	99.2 ± 0.4	34.4 ± 2.4


*^*a*^Samples **2**–**6** were screened at a concentration of 100 μM. Aggregation of Aβ was measured using ThT fluorescence assay; data are reported as the mean ± standard deviation of three separate experiments. Aggregation of tau was measured using ThS fluorescence assay; data are reported as the mean ± standard deviation of three separate experiments. % inhibition was calculated relative to vehicle control (taken as 0% inhibition). ^*b*^No significant effect relative to vehicle control. ^*c*^Experiment repeated using 20.0 μM concentration; statistical significance determined using unpaired two-tailed *t*-test.*

Coffee components **2**–**6** were also tested in the oligomerization assays, and the IC_50_ values for Aβ and α-synuclein are shown in **Table 5**. While components **2**, **3,** and **4** were unable to inhibit the oligomerization of Aβ under the assay conditions, quercetin (**5**) and phenylindane (**6**) were identified as fairly potent inhibitors of Aβ oligomerization with IC_50_ values of 10.3 μM and 42.1 μM, respectively. Oligomerization of α-synuclein was unaffected by **2**, **3,** and **6**, but a pro-aggregation effect for α-synuclein was observed in the presence of caffeic acid (**4**) and quercetin (**5**) at concentrations above 100 μM.

**Table 5:** IC_50_ values of coffee components **2**–**6** in oligomer assays.^a^

Coffee component	Aβ IC_50_ (μM)	α-synuclein IC_50_ (μM)
Chlorogenic acid (**2**)	N.D.	N.D.
Quinic acid (**3**)	N.D.	N.D.
Caffeic acid (**4**)	N.D.	pro-aggregate
Quercetin (**5**)	10.3 ± 1.1	pro-aggregate
Phenylindane (**6**)	42.1 ± 1.4	N.D.


*^*a*^Data are reported as the mean ± standard deviation of three separate experiments. Pro-aggregate effect observed at concentrations >100 μM. N.D. = not determined (>200 μM).*

## Discussion

### Caffeine

The perceived benefits of caffeine (**1**) have been attributed mainly to its action as an A_2A_ receptor antagonist (Svenningsson et al., 1999; Arendash et al., 2009), as the observed neuroprotective effects of caffeine have been replicated using either selective A_2A_ receptor antagonists (Canas et al., 2009) or A_2A_ knockout mice (Chen et al., 2001; Canas et al., 2009). Laboratory experiments have also shown that caffeine treatment can improve working memory and decrease brain Aβ loads in mouse models of AD *via* suppression of both β-secretase (BACE1) and presenilin 1 (PS1)/γ-secretase expression (Arendash et al., 2006, 2009), and improve locomotor function and attenuate dopamine depletion in the 1-methyl-4-phenyl-1,2,3,6-tetrahydropyridine (MPTP) induced toxicity model of PD in mice (Chen et al., 2001; Xu et al., 2010). However, there is still some ambiguity as to whether the neuroprotective effects of coffee are due to the caffeine component: in *Drosophila* models of AD and PD, consumption of caffeinated or decaffeinated coffee led to identical neuroprotective effects whereas pure caffeine failed to provide any therapeutic outcome in this system (Trinh et al., 2010). The authors attribute the benefits of (de)caffeinated coffee to its action on the Nrf2-dependent anti-oxidative pathway, which would protect against neuroinflammation associated with the diseases. Caffeine (**1**) is also known to protect against degeneration of the BBB associated with AD and PD (Chen et al., 2010), and may also contribute to the neuroprotective effect of coffee.

With respect to caffeine’s ability to prevent the aggregation of amyloidogenic proteins in the brain, previous reports have suggested that caffeine (**1**) may inhibit the aggregation of Aβ by forming hydrophobic contacts with monomers or small aggregates (Sharma and Paul, 2016; Sharma et al., 2016). In this system, a modest but observable effect of caffeine on Aβ fibrillization occurs at a caffeine:Aβ ratio of 3:1, with significant levels of inhibition achieved at or above a 10-fold molar excess of caffeine. Our results suggest that any neuroprotective properties of caffeine (**1**) are not due to its association with amyloidogenic proteins or protein aggregates associated with AD, as caffeine failed to alter the aggregation profile of Aβ (50-fold molar excess caffeine) or tau in our fibrillization assays (**Figures 2D** and **3D**). The differences in experimental outcomes are likely due to differences in assay conditions, wherein the prior study was conducted using truncated Aβ_16-22_ (rather than Aβ_1-40_ used in this investigation) and assays were conducted in pure water in an effort to closely replicate the computational simulations performed by the group. This surprising difference in experimental outcomes highlights the complexity of the aggregation process, and speaks to the importance of designing assay conditions which closely mimic physiological conditions to allow translation of experimental results to *in vivo* environments. Changes to the solution pH, buffer composition, concentration of Aβ or tau etc. are known to produce variation in the experimental results (LeVine, 1993; Tiiman et al., 2015), and assay conditions which are most representative of the biological environment are more likely to be reproduced in living systems. However, there are no assurances that *in vitro* results will be replicated *in vivo*, and caution should be exercised during interpretation of *in vitro* data. Higher concentrations of caffeine (**1**) were not investigated given that the coffee extracts displayed more potent aggregation inhibition activity than caffeine at the same concentration (**Figures 2** and **3**, **Tables 1**–**3**).

Caffeine (**1**) also failed to inhibit the oligomerization of α-synuclein, and no pro-aggregation effect was observed (**Table 3**). This result is consistent with a previous report on the fibrillization of α-synuclein in the presence of caffeine (**1**) (Kardani and Roy, 2015): fibrils grown in the presence of caffeine (**1**) showed different morphology relative to fibrils grown in its absence, but the authors claim that this was not due to an interaction of caffeine (**1**) with the α-synuclein protein. The lack of an observable interaction between caffeine (**1**) and Aβ/tau/α-synuclein would suggest that the observed activity of the coffee extracts is not due to the caffeine component. While caffeine (**1**) may have aggregation inhibition activity at higher concentrations, other components found in coffee are likely to be more potent inhibitors of protein aggregation. The comparable activity observed between the caffeinated and decaffeinated coffee extracts support this hypothesis.

### Chlorogenic Acids

Chlorogenic acids (CGAs) are the major polyphenolic acid found in coffee beans, accounting for up to 15% dry weight of green (unroasted) coffee beans. There are nine isomers of CGAs that have been identified, with the major isomer being 5-*O*-caffeoquinolinic acid (**2**) which accounts for approximately 60% of total CGA content (Farah and Donangelo, 2006). A typical cup of coffee contains 70—300 mg CGAs depending on the type of bean and the roasting procedure (Farah and Donangelo, 2006; Ludwig et al., 2014). CGAs are known to enter the bloodstream *via* intestinal absorption (Olthof et al., 2001) and have been correlated to a variety of health benefits due to their anticarcinogenic, antithrombotic, anti-inflammatory, and hypoglycemic effects (Johnston et al., 2003; Feng et al., 2005; dos Santos et al., 2006; Lee and Zhu, 2006; Park, 2009). Given the potential health benefits of CGAs, as well as its bioavailability, we felt that investigation of CGAs was warranted. Being the major CGA component, we tested 5-*O*-caffeoquinolinic acid (**2**) as a representative example of the CGAs found in coffee.

A considerable body of literature has shown that administration of CGAs to various neuronal cell lines *in vitro* can improve cell viability under conditions of oxidative stress or in the presence of neurotoxins (Anggreani and Lee, 2017; Nabavi et al., 2017). The neuroprotective effects were attributed to the high antioxidant capacity of CGAs, which protects cells from damage by reactive oxygen species. It has also been demonstrated that CGA treatment attenuates the pro-inflammatory response of microglia upon lipopolysaccharide (LPS) stimulation (Shen et al., 2012). Our data show that **2** is a potent inhibitor of Aβ fibrillization in the ThT assay at 100 μM (**Figure 4A**), which is in agreement with a previously reported IC_50_ value of ca. 40 μM for inhibition of Aβ fibrillization by **2** (Masuda et al., 2006). However, **2** was unable to inhibit the oligomerization of Aβ (**Table 5**), suggesting that the interaction of **2** with monomeric/oligomeric Aβ proteins is different than its interaction with larger Aβ aggregates. Such an effect has been reported previously, wherein small organic molecules inhibited the formation of larger aggregates of Aβ (Masuda et al., 2006), tau (Taniguchi et al., 2005), and α-synuclein (Dedmon et al., 2005) by stabilizing the smaller oligomeric aggregates. Our data for the interaction of **2** with Aβ are consistent with this type of behavior.

Tau fibrillization was unaffected in the presence of **2** at 100 μM (**Figure 4B**), which is again consistent with literature data (Masuda et al., 2006), and no observable interaction between **2** and α-synuclein could be detected in our oligomerization assay (**Table 5**). However, **2** is reported to be a reasonably potent inhibitor of α-synuclein aggregation, with an IC_50_ for fibrillization inhibition of ca. 80 μM (Masuda et al., 2006). The absence of an observable inhibitory effect of **2** toward α-synuclein oligomerization would again suggest that **2** interacts differently with larger and smaller aggregates of α-synuclein, and that **2** may inhibit α-synuclein fibrillization *via* stabilization of smaller oligomeric species analogous to what is observed for Aβ (Masuda et al., 2006). Given that soluble amyloid oligomers of Aβ, tau and α-synuclein share common structural features (Kayed et al., 2003), it is reasonable to assume that **2** would exhibit similar interaction behaviors toward oligomeric Aβ and α-synuclein which is consistent with our results.

Although CGAs are quite bioavailable it has been demonstrated that compounds such as **2** are unlikely to cross the BBB (Lardeau and Poquet, 2013). As such, CGAs in coffee are not likely to inhibit the aggregation of amyloidogenic proteins in the brain. Secondary mechanisms of neuroprotection cannot be ruled out; ischemic rat models that were administered CGA showed improvements in sensory-motor function and reduced levels of brain compression *versus* a control group (Ahn et al., 2011). Ischemic events have been correlated to AD progression (Kalaria, 2000), and it is possible that consumption of CGAs found in coffee could have neuroprotective effects in spite of the low BBB penetrance. Nonetheless, given that **2** is only able to inhibit the aggregation of Aβ at relatively high concentrations, together with the lack of any observable interaction with tau or α-synuclein, it is unlikely that **2** or other CGAs found in brewed coffee are responsible for the observed aggregation inhibition activity of the coffee extracts.

### Quinic Acid and Caffeic Acid

During the roasting of coffee beans, CGAs are transformed *via* ester cleavage processes into quinic acid (**3**) and various phenolic cinnamates. A typical light roast coffee bean will lose approximately 25% of the initial CGA content, whereas medium and dark roast coffee beans will lose 45 and 90% of their CGA content, respectively (Trugo and Macrae, 1984; Ludwig et al., 2014). The majority of CGA that is “lost” during roasting is incorporated into melanoidans *via* the Maillard reaction. Melanoidins, though indigestible, may serve a prebiotic function by promoting the growth of beneficial types of gut bacteria and acting as dietary fiber (Reichardt et al., 2009), but were omitted from our investigation on the basis that they would not be absorbed into the bloodstream. Caffeic acid (**4**), which is readily bioavailable (Olthof et al., 2001), was chosen as a representative example of the phenolic cinnamates found in brewed coffee, as it is formed *via* ester cleavage of 5-*O*-caffeoquinolinic acid (**2**) [the major CGA component found in coffee beans (Farah and Donangelo, 2006)]. Quinic acid (**3**) was also tested in the protein aggregation assays, although it is worth noting that some free quinic acid (**3**) is converted to the 1,5-lactone during roasting (Farah and Donangelo, 2006). However, **3** displayed no observable activity as an aggregation inhibitor of Aβ, tau or α-synuclein.

Caffeic acid (**4**) is known to have antioxidant properties (Guillot et al., 1996; Gulcin, 2006), and the related ester derivative rosmarinic acid has been shown to inhibit the fibrillization of Aβ, tau and α-synuclen *in vitro* (Masuda et al., 2006). In line with these previous experiments, our assays show that **4** is an effective inhibitor of Aβ fibrillization (**Figure 4A**), yet no inhibitory effect toward Aβ oligomerization was observed (**Table 5**). **4** was also unable to inhibit the fibrillization of tau (**Figure 4B**), the other hallmark protein aggregate of AD. The similar behavior of **2** and **4** toward Aβ and tau would suggest that the observed activity of **2** is due to the hydroxycinnamic acid moiety (i.e., **4**) given that quinic acid (**3**) had no observable inhibitory effect in the aggregation assays. Of note, **4** was observed to induce aggregation of α-synuclein at concentrations above 100 μM (**Table 5**), which could have a neuroprotective effect by promoting Lewy body formation and thus reducing the concentration of toxic oligomer species in the brain (Pinotsi et al., 2016; Ono, 2017). However, the high concentration of **4** required to induce aggregation of α-synuclein is not likely to accumulate *in vivo*.

Despite the capacity of **4** and its derivatives to enter the bloodstream, it has been demonstrated that compounds of this type are not likely to penetrate the BBB (Lardeau and Poquet, 2013). Thus it is unlikely that any neuroprotective effects of coffee are derived from these components. However, a secondary mechanism involving the action of polyphenols on the gut microbiome (Olthof et al., 2001; Johnston et al., 2003) cannot be ruled out, particularly in PD where environmental factors are suggested to contribute to the pathology of the disease (Di Monte et al., 2002; Patel et al., 2005). Changes in hormone secretion and molecular absorption could ultimately lead to less accumulation of neurotoxic compounds in the brain, although further investigation is required to identify such an effect. Nonetheless, given that **3** and **4** did not inhibit the oligomerization of Aβ, and no aggregation inhibition activity was observed for tau, it is unlikely that these coffee components are responsible for the observed activity of the coffee extracts.

### Quercetin

While caffeine and CGAs represent the main components found in coffee beans, a variety of flavonoids have also been identified, albeit in lower concentrations. Quercetin (**5**), a known coffee flavonoid, is reported to have antioxidant properties which help mitigate oxidative stress and inflammation (Guillot et al., 1996; Laura et al., 2011). It has also been demonstrated that **5** can increase glutathione (GSH) expression, providing additional mechanisms of antioxidant/anti-inflammatory neuroprotection (Lee et al., 2016). In fact, it has recently been suggested that quercetin (**5**) rather than caffeine (**1**) is the component which is responsible for the neuroprotective effects of coffee (Lee et al., 2016), although the authors suggest a mechanism based on antioxidant/anti-inflammatory pathways (inhibition of protein aggregation was not investigated in this report). Pharmacokinetic data has shown that **5** and its metabolites accumulate in the brains of rats (Ishisaka et al., 2011) despite the low bioavailability [approximately 20% absorption following oral administration (Chen et al., 2005)].

Consistent with previous reports (Masuda et al., 2006; Matos et al., 2017), we observed that **5** is a potent inhibitor of Aβ aggregation (**Figure 4A**, **Table 5**), yet a loading of 100 μM **5** showed only a modest inhibitory effect for tau fibrillization (**Figure 4B**), again consistent with previous data (reported IC_50_ for tau fibrillization by **5** > 200 μM) (Masuda et al., 2006). However, in contrast to literature data, we observed that **5** was able to promote the aggregation of α-synuclein rather than inhibit its fibrillization (**Table 5**) (Masuda et al., 2006). This discrepancy is likely due to the differences in assay conditions (oligomer assay *versus* fibril assay), which suggests that **5** interacts differently with monomeric/oligomeric α-synuclein and its mature aggregates. Indeed, such effects have been described previously (Zhu et al., 2013); **5** inhibits the formation of α-synuclein fibrils *via* stabilization of smaller oligomeric species which form prior to fibrillization. Covalent modification of the α-synuclein monomer/oligomer by **5** leads to an increase in hydrophilic surface area, stabilizing the α-synuclein-quercetin adduct in aqueous medium and preventing further aggregation. It has also been suggested that **5** may promote the formation of small oligomeric aggregates of Aβ through a similar mechanism, which have the potential to be neurotoxic (Matos et al., 2017). However, no pro-aggregation effect of **5** was observed in our Aβ oligomerization assay (**Table 5**), and in fact **5** was shown to be an effective inhibitor of Aβ oligomerization (IC_50_ = 10.3 μM).

Our data, taken together with literature reports, suggest that quercetin (**5**) in brewed coffee may provide neuroprotection *in vivo* through inhibiting the aggregation of pathogenic Aβ in the brain in addition to its known antioxidant/anti-inflammatory properties (Sabogal-Guaqueta et al., 2015; Lee et al., 2016). However, the inability of **5** to inhibit tau fibrillization limits its efficacy as a therapeutic agent to treat protein misfolding disorders associated with AD. In addition, the propensity of **5** to promote and stabilize oligomeric aggregates of α-synuclein through covalent modification is a potential disadvantage of using **5** to treat PD pathology. It has also been shown that **5** can form smaller oligomeric aggregates of α-synuclein at the expense of larger fibrillar aggregates (Zhu et al., 2013). This is another drawback, as fibril formation is suspected to be neuroprotective.

### Phenylindanes

Phenylindanes are formed from hydroxylated cinnamates such as caffeic acid (**4**) during the roasting of coffee beans (Guillot et al., 1996; Stadler et al., 1996), and mechanisms for their formation have been proposed (Stadler et al., 1996; Frank et al., 2007). Higher concentrations of phenylindanes are found in dark roast coffees (longer roasting times) such as espressos, and are largely responsible for the bitter taste of dark roast coffee blends (Frank et al., 2007). Literature reports have shown that the phenylindanes found in coffee brews display surprisingly potent antioxidant activity (Guillot et al., 1996; Stadler et al., 1996), more potent than 5-*O*-caffeoquinolinic acid (**2**), caffeic acid (**4**) and quercetin (**5**), but their ability to interact with amyloidogenic proteins has not yet been reported. Given the observed differences in activity between the light roast and dark roast coffee extracts (**Tables 2** and **3**) we felt investigation of this coffee component was warranted.

We observed that the phenylindane mixture **6** (1.00:1.87 *cis*:*trans* **6**) displayed potent aggregation inhibition activity in both the Aβ fibrillization assay (**Figure 5A**) and the Aβ oligomerization assay (**Table 5**). Further, **6** displayed more potent levels of tau aggregation inhibition (**Figure 5B**) than any other compound investigated. Given that the dark roast coffee extracts (both caffeinated and decaffeinated) show more potent levels of aggregation inhibition in the Aβ oligomer assays *versus* light roast coffee extract (**Table 3**), and higher quantities of **6** are found in darker roast coffee beans, we propose that it is the phenylindane (**6**) component which is largely responsible for the observed aggregation inhibition activity of coffee extracts for both Aβ and tau. Given that the decaffeination procedure occurs before roasting of the coffee beans, it is reasonable to assume that similar quantities of **6** are produced in both types of dark roast coffees (assuming that roasting times are also similar).

It could be argued that the increase in roasting time from light roast to dark roast coffee beans might also lead to increased quercetin (**5**) concentration in dark roast coffee blends *via* hydrolysis of quercetin glycosides (glycoside hydrolysis may increase the concentration of quercetin aglycone **5**), which might correlate with increased aggregation inhibition activity of dark roast coffee extracts. However, it has been shown that while the concentration of quercetin glycosides decreases under pyrolysis (i.e., roasting) conditions, the concentration of **5** and its derivatives (oxidation products) does not change significantly (Rohn et al., 2007). A rationale is proposed wherein **5** and its derivatives, which are inherently electrophilic, react with nucleophilic components present in the food mixture to generate polymeric composites such as tannins (Rohn et al., 2007; Oracz et al., 2015). More likely, increased roasting of coffee beans would cause a decrease in the amount of **5**. We therefore propose that it is the higher concentration of phenylindane (**6**) in dark roast coffee extracts that is responsible for the increase in aggregation inhibition activity of dark roast coffee extract *versus* light roast coffee extract in the Aβ oligomerization assay (**Table 3**).

When **6** was tested in the α-synuclein aggregation assay, we were unable to calculate an apparent IC_50_ value nor did we observe any pro-aggregation behavior (**Table 5**). The absence of any observable interaction between **6** and α-synuclein would suggest that **6** is not responsible for the pro-aggregation activity of the coffee extracts. More likely, this behavior is derived from caffeic acid (**4**) and quercetin (**5**). While it is unlikely that any neuroprotective effects of coffee with respect to PD is due to the interaction of **6** with α-synuclein, it is plausible that **6** could elicit a neuroprotective effect in PD pathologies by other mechanisms such as neuroinflammatory/antioxidant pathways or by altering gene/protein expression to reduce the amount of pathogenic α-synuclein in the brain. A similar mechanism could also be operative for AD pathologies, although such an effect has yet to be reported in either case. Further research is thus warranted to determine if phenylindanes in coffee promote additional mechanisms of neuroprotection in cell or animal models of AD and PD.

Information on the bioavailability **6** has yet to be reported. It is known that while **6** is water soluble, it can be completely extracted into ether (99.8%) from the water phase (Guillot et al., 1996) giving promise for oral bioavailability and BBB penetrance. Taken together, these results suggest that the phenylindane scaffold is a promising lead for the development of drug-like molecules to treat neurodegenerative disorders, in particular those involving the aggregation of pathogenic Aβ and tau (AD). Further investigation into the bioavailability and pharmacokinetics of phenylindanes in coffee, as well as their BBB penetrance is warranted.

## Conclusion

In summary, we have quantified the aggregation inhibition activity of three instant coffee extracts (light roast, dark roast, decaffeinated dark roast) and a variety of components found in coffee (**1**–**6**) toward Aβ, tau and α-synuclein. We have identified phenylindane (**6**) as a dual-inhibitor of Aβ and tau aggregation, and propose that it is the phenylindane component of coffee that is responsible for the observed aggregation inhibition activity of the coffee extracts toward Aβ and tau. While our investigation did not identify any aggregation inhibition activity of coffee extracts toward α-synuclein, we have identified caffeic acid (**4**) and quercetin (**5**) as pro-aggregates of α-synuclein at concentrations above 100 μM.

While the perceived neuroprotective effect of coffee consumption is likely due to a combination of factors related to the various biological activities of **1**–**6**, the ability of **6** to interact directly with pathogenic Aβ and tau to inhibit their aggregation is noteworthy. Inhibition of Aβ and tau aggregation by phenylindanes (**6**) in coffee can be considered as a plausible mechanism of neuroprotection in AD pathologies for individuals who consume coffee beverages. To our knowledge this is the first report of the aggregation inhibition activity of **6**, and the phenylindane (**6**) scaffold should be considered for future drug discovery projects which target the protein aggregation process. The ability of **6** to reduce Aβ, tau and α-synuclein loads in cell and animal models of AD and PD is currently under investigation by our group, and will be reported in due course.

## Data Availability Statement

The raw data supporting the conclusions of this manuscript will be made available by the authors, without undue reservation, to any qualified researcher.

## Author Contributions

RM performed chemical synthesis and structural characterization, planned biological assays, data interpretation and drafting the manuscript. YW planned biological assays, performed all biological assays and processed all raw data obtained in biological assays. DW helped edit and revise the manuscript.

## Conflict of Interest Statement

The authors declare that the research was conducted in the absence of any commercial or financial relationships that could be construed as a potential conflict of interest.
